# CELLFOOD™ induces apoptosis in human mesothelioma and colorectal cancer cells by modulating p53, c-myc and pAkt signaling pathways

**DOI:** 10.1186/1756-9966-33-24

**Published:** 2014-03-05

**Authors:** Barbara Nuvoli, Raffaela Santoro, Simona Catalani, Serafina Battistelli, Serena Benedetti, Franco Canestrari, Rossella Galati

**Affiliations:** 1 Molecular Medicine Area, Regina Elena National Cancer Institute, Via Elio Chianesi 53, 00144 Rome, Italy; 2 Department of Biomolecular Sciences, Section of Clinical Biochemistry and Cellular Biology, University of Urbino “Carlo Bo”, Via Ubaldini 7, 61029 Urbino, PU, Italy

**Keywords:** CELLFOOD™ (CF), Nutraceutical, Mesothelioma, Colorectal cancer

## Abstract

**Background:**

CELLFOOD™ (CF) is a nutraceutical non-addictive, non-invasive, and completely non-toxic unique proprietary colloidal-ionic formula. Little is known about its effect on cancer cells in solid tumors. The aim of this study was to evaluate the effect that CF has on different cancer cell lines and the mechanism by which the nutraceutical works.

**Methods:**

The effect of CF on HFF (normal fibroblasts), Met5A (mesothelium), MSTO-211H, NCI-2452, Ist-Mes1, MPP89, Ist-Mes2 (mesothelioma), M14 (melanoma), H1650, H1975 (lung cancer), SKRB3 (breast cancer), and HCT-116 (colorectal cancer) cell growth was tested by cell proliferation and clonogenic assay. Among all of them, MSTO-211 and HCT-116 were analyzed for cell cycle by flow cytometry and western blot.

**Results:**

All human cancer lines were suppressed on cell growth upon 1:200 CF treatment for 24 and 48 hours. Death was not observed in HFF and Met5A cell lines. Cell cycle analysis showed an increased sub-G1 with reduction of G1 in MSTO-211 and a cell cycle arrest of in G1 in HCT116. Activation of caspase-3 and cleavage of PARP confirmed an apoptotic death for both cell lines. Increased expression levels of p53, p21, and p27, downregulation of c-myc and Bcl-2, and inhibition of Akt activation were also found in CF-treated MSTO-211 and HCT-116 cells.

**Conclusions:**

These findings ascertained an interaction between p53, c-myc, p21, p27, Bcl-2, PI3K/Akt pathway, and CF-induced apoptosis in MSTO-211H and HCT-116 cells, suggesting that CF acts as an important regulator of cell growth in human cancer cell lines. CF could be a useful nutraceutical intervention for prevention in colon cancer and mesothelioma.

## Background

CELLFOOD™ (CF) is a unique, proprietary concentrate of 78 ionic minerals, 34 enzymes, 17 amino acids, electrolytes, and dissolved oxygen, held in a negatively-charged suspension utilizing deuterium, the only non-radioactive isotope of hydrogen. CF possesses antioxidant properties which protect erythrocytes, lymphocytes, and biomolecules against free radical attacks, suggesting that it may be an adjuvant intervention in the prevention and treatment of various physiological and pathological conditions related to oxidative stress [1]. The oral supplementation of CF for a period of six months significantly improves fibromyalgia symptoms and health-related quality of life of fibromyalgic patients compared to placebo [2]. CF treatment on leukemia cell lines induces cell death due to apoptotic mechanisms and altering cell metabolism through HIF-1α and GLUT-1 regulation [3]. However, the anti-cancer activities and potential anti-cancer mechanisms of the nutraceutical in solid tumors have not yet been elucidated.

Many physiological processes, including proper tissue development and homeostasis, require a balance between apoptosis and cell proliferation. All somatic cells proliferate via a mitotic process determined by progression through the cell cycle. Apoptosis (programmed cell death) occurs in a wide variety of physiological settings, where its role is to remove harmful, damaged or unwanted cells. Apoptosis and cell proliferation are linked by cell-cycle regulators and apoptotic stimuli that affect both processes. A failure in regulating proliferation together with suppression of apoptosis are the minimal requirements for a cell to become cancerous [4].

In the context of aberrant growth control, many important genes responsible for the genesis of various cancers have been discovered and the pathways through which they act characterized. Two proteins involved intimately in regulating cell proliferation are Akt and the tumor suppressor p53 (p53). The protein serine/threonine kinase Akt (also known as protein kinase B or PKB) plays an important role in averting cell death. A diverse range of physiological stimuli induce Akt kinase activity, including many trophic factors which promote survival, at least in part, through Akt activation via the phosphatidylinositide 3′-OH kinase (PI3K) signaling cascade. Moreover, induced Akt activity (p-AKT) (due to overexpression) is sufficient to block apoptosis triggered by many death stimuli [5]. p53 has an important protective role against undesired cell proliferation. As such, p53 has been described as the “guardian of the genome”. The p53 protein is a transcription factor that normally inhibits cell growth and stimulates cell death in response to myriad stressors, including DNA damage (induced by either UV or chemical agents such as hydrogen peroxide), oxidative stress, and deregulated oncogene expression [6-10].

p53 activation is characterized by a drastic increase and its rapid accumulation in stressed cells [11]. p53 is a master gene regulator controlling diverse cellular pathways, by either activating or repressing downstream genes. Among such genes, there is also the proto-oncogene c-*myc*, which is negatively regulated by p53 [12]. The c-*myc* proto-oncogene encodes the c-myc transcription factor, and was originally identified as the cellular homologue to the viral oncogene (v-*myc*) of the avian myelocytomatosis retrovirus [13,14]. More recently, elevated or deregulated expression of c-myc has been detected in a wide range of human cancers, and is often associated with aggressive, poorly differentiated tumours [15,16]. One of the key biological functions of c- myc is its ability to promote cell-cycle progression [17-19] by repressing genes as the cyclin-dependent kinase inhibitors p21/WAF1 (p21) and p27Kip1 (p27), which are involved in cell-cycle arrest [20-22]. Cell division relies on the activation of cyclins, which bind to cyclin-dependent kinases to induce cell-cycle progression towards mitosis. Following anti-mitogenic signals, p21 and p27 bind to cyclin-dependent kinase complexes to inhibit their catalytic activity and induce cell-cycle arrest [23].

Acceleration of tumorigenesis is observed when apoptosis is suppressed by overexpression of anti-apoptotic proteins such as Bcl2 [24]. When anti-apoptotic Bcl-2 family members are overexpressed, the ratio of pro- and anti-apoptotic Bcl-2 family members is disturbed and apoptotic cell death can be prevented. Targeting the anti-apoptotic Bcl-2 family of proteins can improve apoptosis [25-27]. Apoptosis induction is arguably the most potent defence against cancer growth. Evidence suggests that certain chemopreventive agents can trigger apoptosis in transformed cells *in vivo* and *in vitro*, which appears to be associated with their effectiveness in modulating the process of carcinogenesis.

In this study, we analyzed the effect of CF on 12 different cell lines showing that the nutraceutical has anti-cancer activity. Among all, colon cancer (HCT-116) and mesothelioma (MSTO-211H) cell lines were the most sensitive and were selected to study the action of CF on cancer. The nutraceutical treatment induced death by apoptosis, upregulation of p53 and downregulation of c-myc, pAkt, and Bcl-2. Given the central role of these molecular targets in cell proliferation and death, the potential preventive benefits of CF in human cancers are self-evident.

## Methods

### Cell culture

Breast (SKRB3), colorectal (HCT116), lung (H1650, H1975), melanoma (M14), mesothelioma (MSTO-211H, NCI-2452, Ist-Mes1, MPP89, Ist-Mes2) cancer cell lines, and fibroblast (HFF) and mesothelio (MeT5A) cell lines were gradually conditioned in DMEM/F12 + Glutamax (Invitrogen Life Technologies, Paisley, UK) supplemented with 10% FBS and antibiotics and maintained at 37°C and 5% CO2.

### Cellfood

CF (liquid) was kindly provided by Eurodream srl (La Spezia, Italy) and stored at room temperature. CF was diluted in phosphate buffered saline (PBS) and sterilized using a 0.45 μm syringe-filter before use.

### Cell growth assays

For cell growth experiments, cells were plated in quintuplicates in 96-well culture plates (Nunc, Milan, Italy) at a density of 3 × 10^3^ cells/well. 24 h later, the medium was replaced with fresh growth medium containing 1:200, 1:400, 1:800, 1:1600 dilutions of CF. At 24 and 48 h of treatment, XTT labelling reagent (final concentration 0.5 mg/ml) was added to each well, and the samples were incubated for an additional 4 h at 37°C. The XTT assay (Cell proliferation Kit (XTT), Roche Molecular Biochemicals, Indianapolis, IN) is based on the cleavage of the yellow tetrazolium salt XTT to form an orange formazan dye by metabolic active cells. Absorbance was measured at 492 nm with a reference wavelength at 650 nm and the absorbance values of treated cells were presented as a percentage of the absorbance versus non treated cells (CNTRL). All experiments were repeated three times.

The anti-proliferative CF activity was assessed in monolayer cell culture conditions by plating cell lines in a T25 flask. After 24 h, CF (5 μl per ml of medium corresponding to a 1:200 dilution) was added for the time indicated in the experiments. Nothing else was added in CNTRL. The expansion of cell culture proliferation was quantified by manual cell counting. Experiments were repeated in triplicate and media values were calculated.

### Clonogenic assay

Five hundred viable cells per well (treated with CF and CNTRL) were plated in a 35 mm dish and allowed to grow in normal medium for 10-14 days and then stained for 30 min at room temperature with a 6% glutaraldehyde, 0.5% crystal violet solution. Pictures were captured digitally. All experiments were repeated at a minimum twice for each cell line.

### Flow cytometry

For cell cycle analyses, cells were fixed in 70% ethanol and stored at -20°C over night. Fixed cells were treated with 1 mg/ml RNase A (cat. 12091021, Invitrogen Life Technologies, Paisley, UK) for 1 h at 37°C and DNA was stained with Propidium Iodide (Sigma, St. Louis, MO, USA). Samples were acquired with a Guava EasyCyte 8HT flow cytometer (Merck Millipore Billerica, Massachusetts, USA). Cell cycle distribution was shown.

### Western blot analysis

Briefly, 25-50 μg of proteins extracted as described previously from cultured cells [21] were separated by SDS-PAGE and transferred onto nitrocellulose membranes. Membranes were blocked and blotted with relevant antibodies: Bcl-2, p21, p27, p53, c-myc, caspase-3 (Santa Cruz Biotechnology, Santa Cruz, CA, USA), p-AKT, AKT, PARP (Cell Signaling Technology, Danvers, MA) and γ-tubulina (Sigma, Saint Louis MO, USA). Goat anti-mouse or rabbit or goat IgG horseradish peroxidase conjugated secondary antibodies (1:3,000) (Bio-Rad Laboratories; Hercules, CA, USA) were visualized with enhanced chemiluminescence reagent (ECL, Amersham-Pharmacia, Uppsala, Sweden).

## Results

### CF induces death in human cancer cell lines

**Figure 1:**
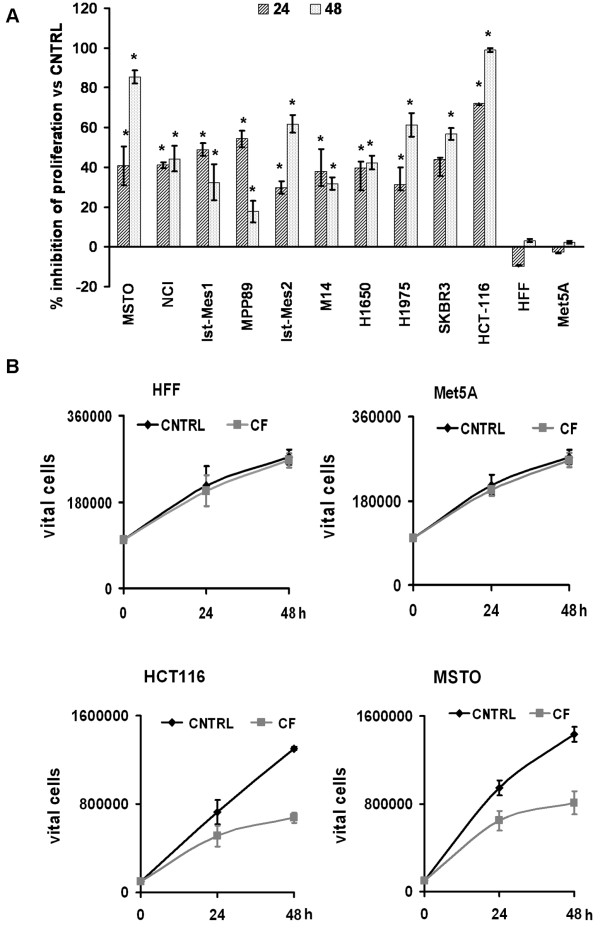
**Effects of CF on cancer and normal human cells. (A)** Cells were cultured in the presence or absence of CF at the 1:200 dilution for 24 and 48 hours. Cell viability was measured using the XTT assay and expressed as% of inhibition of proliferation versus non treated cells (CNTRL). Data are expressed as mean ± SD of at least three independent experiments. * p < 0.05 vs CNTRL. **(B)** HFF, Met5A, HCT-116 and MSTO cells were treated with CF (5 μl/ml, corresponding to a 1:200 dilution) or not (CNTRL) for 24 and 48 hours, the graphs represent the vital cells number measured by manual count. Data are expressed as mean ± SD of at least three independent experiments.

The antiproliferative effect of CF dilutions (1:200, 1:400, 1:800 and 1:1600) was assessed by Cell proliferation kit upon 24 and 48 h of treatment was tested on different cell lines (Table 1). In all cancer cell lines CF had a dose-response effect, in fact, the slight reduction in the proliferative activity at 1:800 dilution increased and became significant at 1:200 dilution. At this dilution dose, no significant changes in the HFF and Met5A cell lines were observed (Figure 1A). HCT-116 and MSTO-211 were the most sensitive to CF and for this reason they have been selected for further studies. By manual count of vital cells, the absence of inhibition of cell growth in HFF and Met5A and the antiproliferative activity in HCT-116 and MSTO-211 upon CF treatment were confirmed (Figure 1B) although with different percentages compared to those obtained with the proliferation kit. This shows that CF inhibits the proliferation of cancer cell lines.

**Table 1:** Cell lines tested with CF

**Name**	**Source**
H1650	Lung cancer
H1975	Lung cancer
HCT-116	Colon cancer
HFF	Fibroblasts^**§**^
Ist-Mes1	Mesothelioma
Ist-Mes2	Mesothelioma
M14	Melanoma
Met-5A	Mesothelium^**§**^
MPP89	Mesothelioma
MSTO-211H	Mesothelioma
NCI-H2452	Mesothelioma
SKBR3	Breast cancer

Normal^**§**^ and cancer cell lines.

### CF reduces the clonogenic survival of MSTO-211 and HCT-116 cell lines

**Figure 2:**
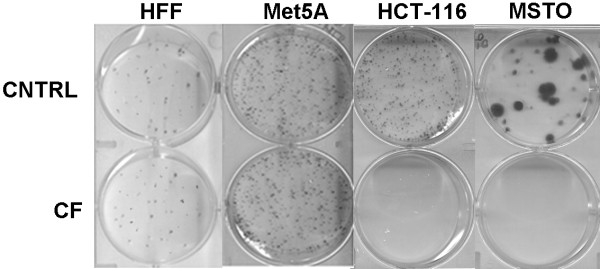
**HFF, Met5A, HCT116 and MSTO colony formation capacity upon CF treatment.** Five hundred viable cells, pretreated for 48 h with CF (1:200) and CNTRL, were allowed to grow in normal medium for 10-14 days and then stained by crystal violet solution. The image is representative of three independent experiments.

The effects of CF on HCT-116 and MSTO-211 cancer cells and HFF and Met-5A normal cells in clonogenic assays were evaluated. The clonogenic cell survival assay determines the ability of a cell to proliferate indefinitely, thereby retaining its reproductive ability to form a large colony or a clone. This cell is then said to be clonogenic. Single cells were plated and cultured for 10 days with CF 1:200 (Figure 2). Colony formation was absent in HCT-116 and MSTO-211, while HFF and Met-5A colony yields were unaffected. This shows that CF selectively inhibits the ability of HCT-116 and MSTO-211to grow into a colony.

### CF induces apoptosis in HCT-116 and MSTO-211 cell lines

**Figure 3:**
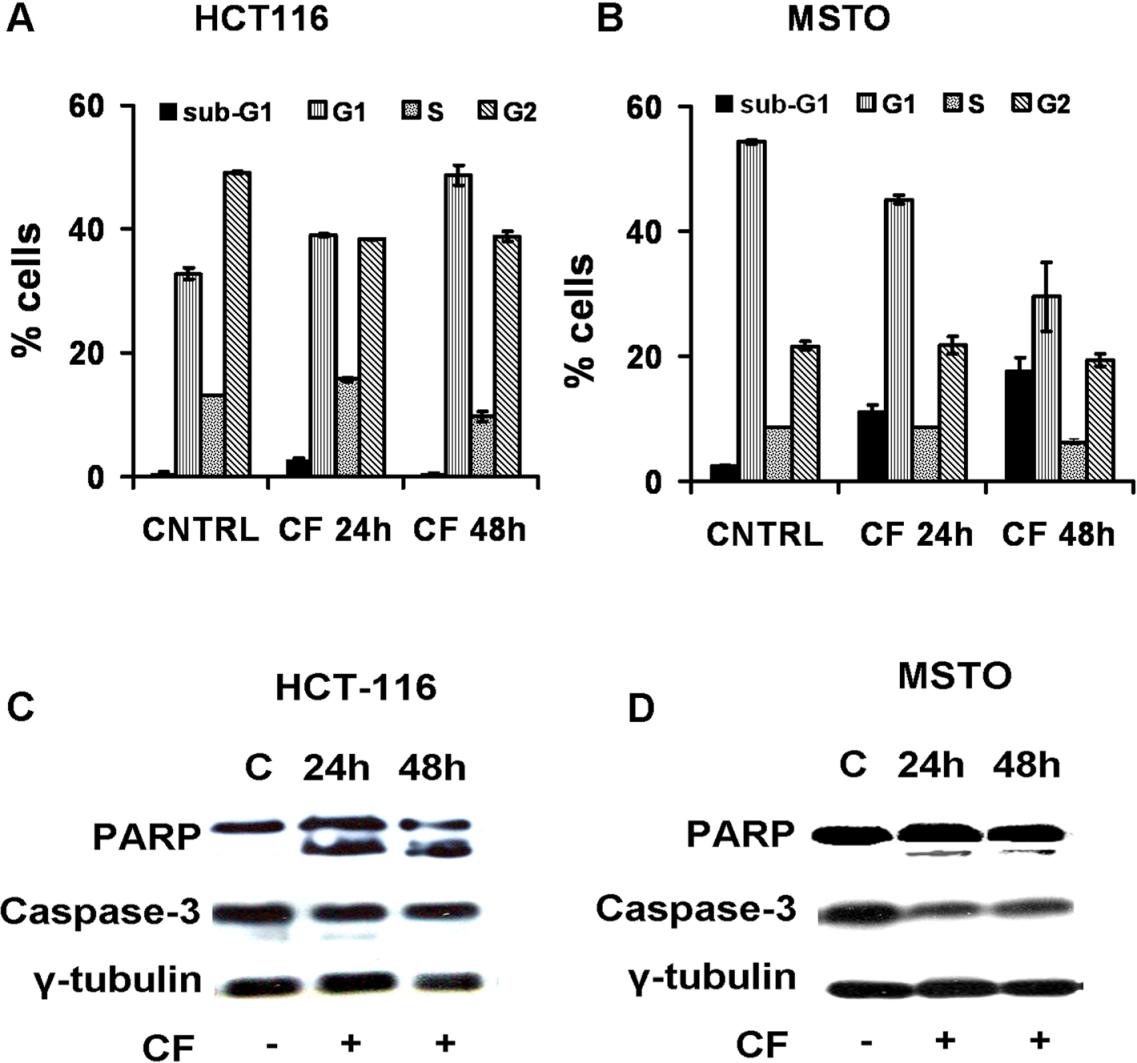
**Effects of CF on the HCT116 and MSTO cell-cycle progression and apoptosis.** Cell cycle analysis after propidium iodide staining was performed by flow cytometry in HCT-116 and MSTO cells untreated (CNTRL) or treated with CF (1:200) for 24 and 48 h (CF24 h and CF48 h). The percentages of HCT-116 and MSTO cells in the different phases of cell cycle was reported in graph **(A)** and **(B)**, respectively. Data are expressed as mean ± SD of at least three independent experiments. Western blot of total lysates indicates that the CF activates caspase-3 and PARP cleavage in HCT-116 **(C)** and MSTO **(D)** cells upon CF treatment (1:200) for 24 and 48 h versus the untreated control **(C)**. γ tubulin was examined as a loading control. The image represents three independent experiments.

In order to confirm whether CF-induced growth inhibition was due to apoptosis, CF-treated and untreated HCT-116 and MSTO-211 cells were analyzed by flow cytometry. The G1 peak was increased in CF-treated HCT-116 cells. The percentage of G1 peak in control and CF-treated HCT-116 cells for 24 and 48 hours was 32.8 ± 0.8, 39.0 ± 0.19 and 48.6 ± 1.5, respectively (Figure 3A). The sub-G1 peak, which is indicator of apoptosis, was raised following 24 and 48 hours of CF-treated MSTO-211 cells. The percentage of this sub-G1 peak in control and CF-treated MSTO-211 cells for 24 and 48 hours was 2.5 ± 0.03, 11.2 ± 1.0 and 17.8 ± 2.0, respectively (Figure 3B), thereby suggesting apoptotic cell death. Caspase-3 is expressed in cells as an inactive precursor from which the subunits of the mature caspase-3 are proteolytically generated during apoptosis. In our experiments we used a mouse monoclonal antibody raised against the full length caspase-3, so the reduction of the expression of caspase-3 indicates apoptosis. Expression of caspase-3 and cleavage of poly (ADPribose) polymerase (PARP) (the substrate of caspase-3, an early index of apoptosis) were detected in western blot (Figure 3C,D) in CF-treated HCT-116 and MSTO-211cells. These results show that CF induces apoptosis in HCT-116 and MSTO-211 cells. These results show that CF induces apoptosis in HCT-116 and MSTO-211 cells.

### CF induces apoptosis via upregulation of p53, p21 and p27 and downregulation of c-myc

**Figure 4:**
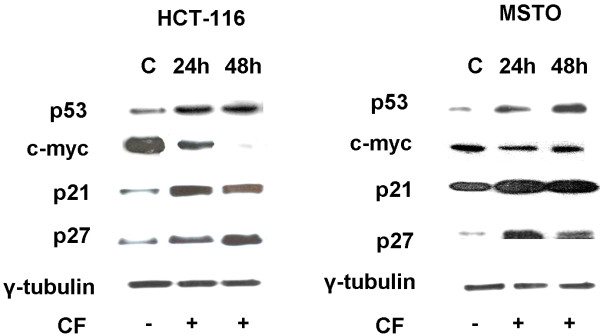
**Expression of p53, c-myc, p21 and p27 in HCT-116 and MSTO cells.** Cells were cultured in the absence or presence of CF (1:200) for the indicated time and whole cell lysates were analyzed by western blot. Data representing three independent experiments with similar results, indicate an upregulation of p53, p21 and p27 and a downregulation of c-myc in HCT-116 and MSTO cell upon CF treatment vs untreated cells. γ tubulin was examined as a loading control.

To clarify the detailed mechanisms underlying CF-induced cell apoptosis, we detected the expression of apoptosis related proteins in CF-treated HCT-116 and MSTO-211cells by western blot assay for the indicated time (Figure 4). We found that the treatment with CF increased the expression of p-53 and of the cell cycle-regulatory proteins p21 and p27 as compared to CNTRL. p53 controls some genes including *c-myc*. By investigating c-myc, we found that its expression is downregulated in CF-treated cells as compared to the control, suggesting that p53 negatively regulates c-myc. There are reports in the literature supporting our findings showing that apoptosis could be induced through downregulation of c-myc in curcumin treated cancer cells [28-30]. These data indicate that p53, c-myc, p21 and p27 play a decisive role in CF-induced apoptosis of HCT-116 and MSTO-211 cells.

### CF induces apoptosis through inhibition of the PI3K/Akt and Bcl-2 signaling pathway

**Figure 5:**
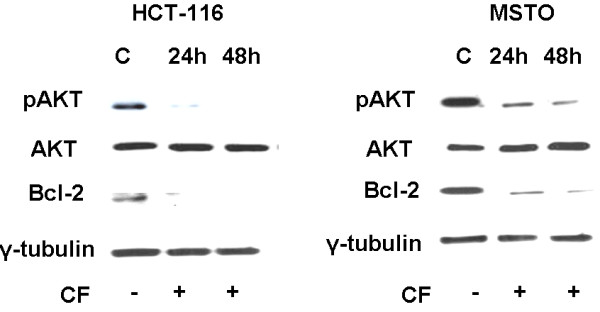
**Effects of CF on the survival pathway in HCT-116 and MSTO cells.** Cells were cultured in the absence or presence of CF (1:200) for the indicated times and whole cell lysates were analyzed by western blot. Data representing three independent experiments with similar results, indicate a downregulation of Bcl-2 and p-AKT, whereas total AKT does not change in HCT-116 and MSTO treated with CF for 24 and 48 h vs untreated cells. γ tubulin was examined as a loading control.

We investigated the effect of CF on PI3K/Akt and Bcl-2 survival pathways. To test the status of Akt activation, the phosphorylation of Akt was measured in HCT-116 and MSTO-211 by western blot analysis (Figure 5). A high level of basal phosphorylated Akt (p-Akt) was observed in both cells, and total Akt levels were found to be almost equal in HCT-116 and MSTO-211 cells. Consequently, we examined the protein expression and phosphorylation level of p-Akt after CF treatment for the indicated times in HCT-116 and MSTO-211 cells. The levels of p-Akt significantly decreased following treatment with CF while total Akt levels did not change (Figure 5). Our experiments on Bcl-2 western blot assay in non-treated and CF-treated HCT-116 and MSTO-211 cells showed an evident decrease of Bcl-2 in CF-treated cells (Figure 5). These data indicate that CF play a decisive role in the survival pathway inhibition in HCT-116 and MSTO-211 cells.

## Discussion

Cancer chemoprevention using natural or synthetic compounds to prevent or suppress the development of cancer is an area of active investigation. Many compounds belonging to diverse chemical classes have been identified as potential chemopreventive agents, including dietary constituents, nutraceuticals, naturally occurring phytochemicals, and synthetic compounds. Because of their safety and the fact that they are not perceived as 'medicine’, natural compounds have created high interest for their development as chemopreventive agents that may find widespread, long-term use in populations at normal risk. Chemopreventive agents function by modulating processes associated with xenobiotic biotransformation, with the protection of cellular elements from oxidative damage, or with the promotion of a more differentiated phenotype in target cells [31-34]. They induce apoptosis, inhibit cellular proliferation, affect angiogenesis and cell metabolism in various cancers, all of which are hindrances to tumor growth [35-37].

It is know that cancer cells can not grow in a high oxygen environment and that the prime cause of cancer is the replacement of the normal oxygen respiration by an anaerobic (without oxygen) cell respiration, focusing the vital importance of oxygen [38]. Our body uses oxygen to metabolize food and to eliminate toxins and waste through oxidation. Cells undergo a variety of biological responses when placed in hypoxic conditions, including switch in energy metabolism from oxidative phosphorylation to glycolysis and activation of signaling pathways that regulate proliferation, angiogenesis and death. Cancer cells have adapted these pathways, allowing tumours to survive and even grow under hypoxic conditions, and tumour hypoxia is associated with poor prognosis and resistance to therapy [39,40]. In most solid tumours, the resistance to cell death is a consequence of the suppression of apoptosis (dependent on mitochondrial energy production).

In this context, CELLFOOD™, the “physiological modulator” aimed to make available oxygen “on-demand” with marked antioxidant effects [1,41,42], was investigated for apoptosis and cancer prevention. CF (also known as Deutrosulfazyme™), is a nutraceutical supplement whose constituents, including 78 trace elements and minerals, 34 enzymes, 17 amino acids, electrolytes and deuterium sulphate, are all naturally occurring substances which are essential to the body’s biochemical functions. We tested the activity of CF on 12 different cell lines, 2 normal and 10 cancerous. Our results showed that CF reduced cell proliferation in a dose-dependent manner in all the cancer cell lines used. Mesothelioma (MSTO-211) and colon cancer (HCT-116) were the most sensitive cell lines to the nutraceutical. Mesothelioma (MM), which commonly originates from mesothelial cells lining the pleural cavity, is an aggressive tumour that is difficult to treat [43]. The number of MM patients is predicted to increase because of the long latency of the disease and historical exposure to asbestos [44]. Colorectal cancer is a major cause of morbidity and mortality throughout the world [45]. CF suppresses cell growth by apoptosis in MSTO-211 and HCT-116 cell lines. In particular, we found that CF caused an increase of sub-G1 and a reduction of G1 in MSTO-211, and a cell cycle arrest in G1 in HCT116. We speculated that CF-induced proliferative block was irreversible due to the significant increase in population with a sub-G1 and G1 DNA content (that are indicative of apoptosis) observed in the treated cells as compared to the untreated ones.

Evidence of apoptosis in MSTO-211 and HCT-116 cells on CF treatment was observed in western blot. CF induces apoptosis by a caspase-dependent pathway. Among the caspase family members, caspase-3 is known to be one of the key executioners of apoptosis because caspase-3 activation causes the cleavage or degradation of downstream important substrates, like PARP, which is the hallmark of caspase-dependent apoptosis. In our experiments, caspase-3 activation and PARP cleavage were detected in CF-treated MSTO-211 and HCT-116. Thus, apoptosis induction by CF was also confirmed by these observations. Nevertheless, to further explain the precise mechanism of CF-induced apoptosis in cancer cells, we examined the expression levels of p53, c-myc, Bcl-2, pAkt and Akt. We identified p53 as the target of CF. p53 is one of the most important tumour suppressor genes, and it is frequently inactivated in various cancers. p53 modulates various cellular functions, such as apoptosis and cell cycle arrest via transcriptional regulation. Interestingly, wild-type p53 expression was detected in 47% of colorectal adenocarcinomas [46], and approximately 70–80% of mesothelioma cells, although having the wild-type p53 gene, show a homologous deletion at the INK4A/ARF locus containing the p14ARF and the p16INK4A genes, which consequently leads to decreased p53 functions despite the wild-type genotype [47]. MSTO-211 and HCT-116 cell lines endowed wild-type p53 and CF treatment increased the expression level of p53.

Accumulating evidence indicates that c-myc has an important function in cell proliferation and apoptosis induction [48]. c-Myc expression is low in quiescent normal cells whereas it is elevated in a broad range of human cancers, such as the malignant pleural mesothelioma, indicating its key role in tumour development [49]. Human malignant pleural mesothelioma shows elevated c-myc expression and it is a transcription factor mediating cancer progression, highly overexpressed in 60% of colorectal cancer, indicating that c-myc is a hallmark of tumorigenesis [50-52]. Studies using conventional c-*myc* transgenic mice, in which the oncogene is constitutively expressed in a given cell type by means of a tissue-specific promoter, have supported the view that deregulated c-*myc*, as an initial event, is important for the formation of certain cancers, albeit with a long latency [24,53,54]. C-myc has also been reported to promote cell cycle re-entry and proliferation through repression of p21 and p27 expression [55]. In our experiments, CF induced an upregulation of p21 and p27 thus, the suppression of c-myc expression by the nutraceutical may render substantial therapeutic benefits in colorectal cancer and mesothelioma patients by inhibiting the driving activities of c-myc in cell proliferation and cell cycle progression.

The phosphatidylinositol 3-kinase (PI3K)/AKT signaling pathway plays an important role in survival when cells are exposed to various kinds of apoptotic stimuli [56,57]. Recent reports have indicated that the activation of Akt pathway is implicated in conferring resistance to conventional chemotherapy and multiple chemotherapeutic agents on cancer cells [58,59]. Akt is hyperactivated in a wide range of human tumours as a result of constitutive activation of growth receptors, mutation of PI3K, and inactivation or loss of PTEN phosphatise [60]. One mechanism by which Akt prevents apoptosis is considered to proceed through phosphorylation and inactivation of the pro-apoptotic protein and also induction of the anti-apoptotic Bcl-2 protein expression [5,61]. The pro-survival Bcl-2 family members are pivotal regulators of apoptotic cell death; therefore, they are considered as attractive targets for drug design [62,63]. Interestingly, we found p-AKT and Bcl-2 downregulation in HCT-116 and MSTO-211 upon CF treatment, thus leading us to believe that CF can be used for the prevention of tumours and can possibly sensitize cancer cells to standard therapy.

## Conclusion

Taken together, these findings establish an interaction between p53, c-myc, Bcl-2, p21, p27 and PI3K/Akt pathway and CF-induced apoptosis in MSTO-211 and HCT-116 cells, which may improve prevention outcomes for mesothelioma and colon cancer. Given the central role of p53, c-myc, Akt and Bcl2 in cell proliferation and death of many cancers, together with the evidence obtained on MSTO-211 and HCT-116 cell lines treated with CF, we believe in the potential chemopreventive benefits of CF in human cancers. Although further investigation is underway in our laboratory, this present work suggests that CF can sensitize cancer cells to standard therapy. In addition, as a nutritional supplement, CF can improve the quality of life of cancer patients undergoing antineoplastic therapy.

## Abbreviations

CF: Cellfood™; GLUT-1: Glucose transporter 1; HIF-1α: Hypoxia inducible factor 1 alpha; MM: Mesothelioma; p53: Tumor suppressor p53.

## Competing interests

The authors confirm that there are no conflicts of interest.

## Authors’ contributions

BN carried out the majority of the experiments. RS contributed to the FACS analysis. SC, SBa, SBe and FC contributed to interpretation of data and study coordination. RG performed the study design, data acquisition and analysis, and manuscript writing. All authors read and approved the final manuscript.
