# Correction: CELLFOOD™ induces apoptosis in human mesothelioma and colorectal cancer cells by modulating p53, c-myc and pAkt signaling pathways

**DOI:** 10.1186/s13046-022-02498-9

**Published:** 2022-09-30

**Authors:** Barbara Nuvoli, Raffaela Santoro, Simona Catalani, Serafina Battistelli, Serena Benedetti, Franco Canestrari, Rossella Galati

**Affiliations:** 1 Molecular Medicine Area, Regina Elena National Cancer Institute, Via Elio Chianesi 53, 00144 Rome, Italy; 2 Department of Biomolecular Sciences, Section of Clinical Biochemistry and Cellular Biology, University of Urbino “Carlo Bo”, Via Ubaldini 7, 61029 Urbino, PU Italy

**Correction: J Exp Clin Cancer Res 33, 24 (2014)**

**https://doi.org/10.1186/1756-9966-33-24**

Following publication of the original article [1], the author identified an error in Fig. 2, specifically:

- Figure 2: clonogenic assays

**Fig. 2:**
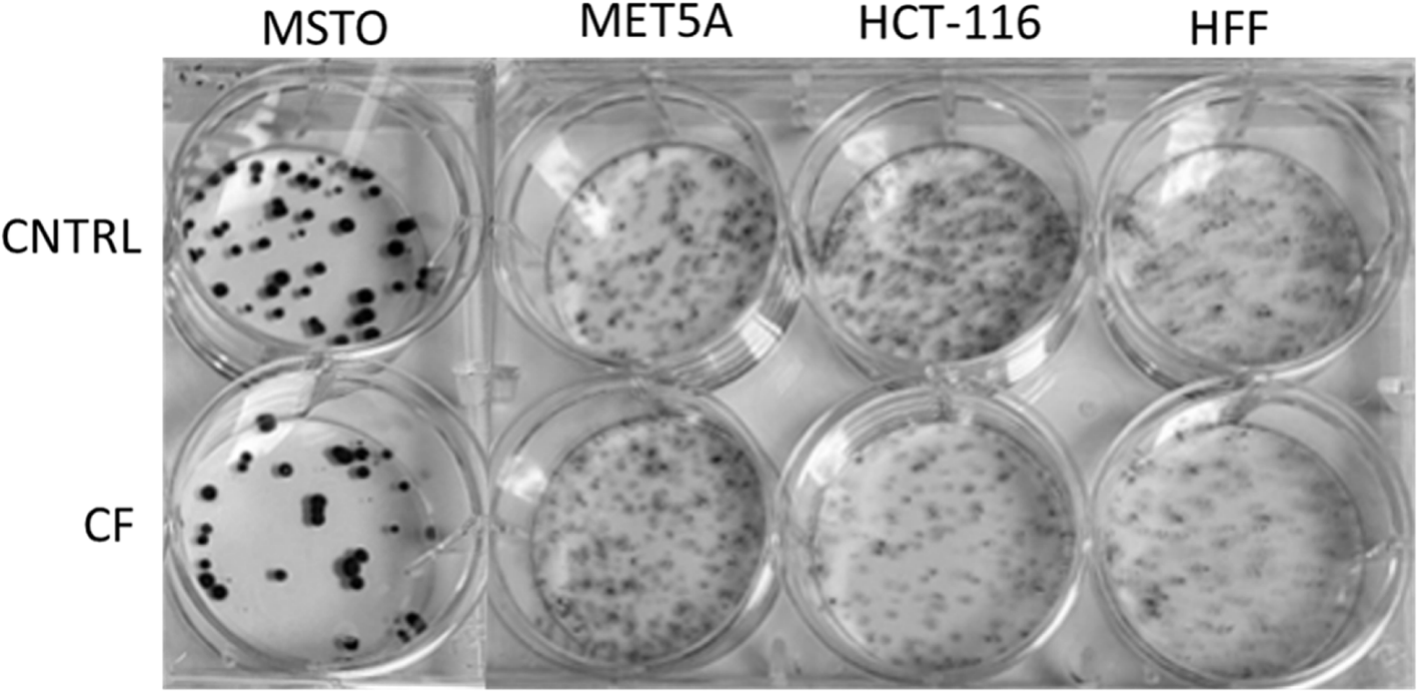
MSTO, Met5A, HCT-116 and HFF colony formation capacity upon CF treatment. Five hundred viable cells, pretreated for 48 h with CF (1:200) and CNTRL, were allowed to grow in normal medium for 10–14 days and then stained by crystal violet solution. The image is representative of three independent experiments

Furthermore, the sentence ‘Colony formation was absent in HCT-116 and MSTO-211, while yields of HFF and Met-5A colonies were not affected.’ under CF reduces the clonogenic survival of MSTO-211 and HCT-116 cell lines of Results section should be updated to ‘Colony formation was reduced in HCT-116 and MSTO-211, while yields of HFF and Met-5A colonies were not affected.’

This correction does not change the result, interpretation, and conclusions of the study.
